# Antagonism of cannabinoid receptor 2 pathway suppresses IL-6-induced immunoglobulin IgM secretion

**DOI:** 10.1186/2050-6511-15-30

**Published:** 2014-06-09

**Authors:** Rentian Feng, Christine A Milcarek, Xiang-Qun Xie

**Affiliations:** 1 Department of Pharmaceutical Sciences, Computational Chemical Genomics Screening Center, University of Pittsburgh School of Pharmacy, Pittsburgh, PA, USA; 2 Department of Immunology, University of Pittsburgh School of Medicine, Pittsburgh, PA, USA; 3 Pittsburgh Drug Discovery Institute, University of Pittsburgh, Pittsburgh, PA, USA; 4 University of Pittsburgh Cancer Institute, Clinical and Translational Science Institute, Pittsburgh, PA, USA

**Keywords:** B cells, IgM, Cannabinoid receptor 2, Liands, STAT3

## Abstract

**Background:**

Cannabinoid receptor 2 (CB2) is expressed predominantly in the immune system, particularly in plasma cells, raising the possibility that targeting the CB2 pathway could yield an immunomodulatory effect. Although the role of CB2 in mediating immunoglobulin class switching has been reported, the effects of targeting the CB2 pathway on immunoglobulin secretion *per se* remain unclear.

**Methods:**

Human B cell line SKW 6.4, which is capable of differentiating into IgM-secreting cells once treated with human IL-6, was employed as the cell model. SKW 6.4 cells were incubated for 4 days with CB2 ligands plus IL-6 (100 U/ml). The amount of secreted IgM was determined by an ELISA. Cell proliferation was determined by the ^3^H-Thymidine incorporation assay. Signal molecules involved in the modulation of IgM secretion were examined by real-time RT-PCR and Western blot analyses or by using their specific inhibitors.

**Results:**

We demonstrated that CB2 inverse agonists SR144528 and AM630, but not CB2 agonist HU308 or CB1 antagonist SR141716, effectively inhibited IL-6-induced secretion of soluble IgM without affecting cell proliferation as measured by thymidine uptake. SR144528 alone had no effects on the basal levels of IgM in the resting cells. These effects were receptor mediated, as pretreatment with CB2 agonist abrogated SR144528-mediated inhibition of IL-6 stimulated IgM secretion. Transcription factors relevant to B cell differentiation, Bcl-6 and PAX5, as well as the protein kinase STAT3 pathway were involved in the inhibition of IL-6-induced IgM by SR144528.

**Conclusions:**

These results uncover a novel function of CB2 antagonists and suggest that CB2 ligands may be potential modulators of immunoglobulin secretion.

## Background

The cannabinoid 1 (CB1) receptor is expressed mainly in the central nervous system and mediates cannabinoid neurobehavioral effects, whereas the CB2 receptor is predominantly expressed in many types of immune cells, such as B and T lymphocytes and monocytes. The CB2 receptor is thought to be the mediator of cannabinoid-induced immunomodulatory effects [1-3]. Due to the distinct expression patterns of CB1 and CB2 in the brain *versus* immune system, public concerns that compounds with high affinity binding to the CB1 subtype may illicit severe psychotropic side effects has overclouded the clinical development [4]. Consequently, research and development of compounds with high CB2 selectivity, predictably with no or diminished psychotropic effects, have garnered much attention particularly in immunomodulation, inflammation, cancer and bone disease prevention and their treatment [5-12].

The mechanisms by which cannabinoid receptors modulate immune function have not been fully elucidated. As an inhibitory G_i/o_ protein-coupled receptor, CB2 activation is associated with the inhibition of cyclic AMP formation, which results from G_i_ protein-induced inhibition of adenylyl cyclase. Conversely, CB2 antagonist SR144528 alone can stimulate the forskolin-sensitive adenylyl cyclase activity, thereby mitigating the inhibition of forskolin-stimulated cAMP [13]. Although the CB1 pathway may also be involved in immunoregulation [14], CB2 has been shown to be the cannabinoid receptor primarily responsible for the anti-inflammatory and possible immune therapeutic effects of cannabis [8,15]. Among the various immune mechanisms influenced by cannabinoids, T helper (Th) cell biasing has been reported with suppression of Th1 (e.g. decrease in IgG2a) and enhancement of Th2 immunity (e.g. increase of serum IgE or IgG1) [8]. Previous studies have shown that delta-9-tetrahydrocannabinol inhibits the mouse plaque-forming cell assay for antibody formation [7]. Furthermore, CB2 mediates immunoglobulin class switching from IgM to IgE in cultures of murine B lymphocytes [16].

A consideration in the study of CB2 and immune function is the model species used. While mouse is the primary animal model for biological studies, one should be cautious to extrapolate human effects from animal data when investigating pharmacological and immunological responses of CB2 ligands in diverse species [15]. Unlike for CB1, there is a considerable level of sequence variation and gene expression difference for CB2 among human, mouse and rat species. Of note, it is the C-terminus of CB2 that plays a critical role in regulating receptor desensitization and internalization [17]. Human B cells express one CB2 transcript while mouse B cells express three CB2 transcripts [18]. Furthermore, the heterogeneity of mouse splenic B lymphocytes may hinder the molecular analysis of the mechanism of action of SR144528 on B cell differentiation [19,20].

Although CB2 is more highly expressed in B cells than in other immune cell subsets, the mechanism by which CB2 regulates B cell function is unclear. Information on the modulatory activity of CB2 ligand SR144528 in the differentiation of B lineage plasma cells is also limited. To explore the role of CB2 receptor signaling in the immunoglobulin production in plasma cell, we employed the human B cell line SKW 6.4 to investigate the effects of CB2 ligands on cytokine-induced IgM production. This cell line has been shown to be capable of differentiating into IgM-secreting cells once treated with human IL-6 [21] and suitable for the analysis of immunomodulator activities [19,22]. Meanwhile, this study also helps us probe the possible therapeutic implication of use of CB2 ligands for certain clinical conditions including hyperimmunoglobulinia. The roles of immunoglobulin relevant transcription factors including Bcl6, PAX5 and XBP-1 as well as STAT in this regulation were also discussed. To our knowledge, this is the first report showing the modulation effects of CB2 antagonists on IL-6-induced IgM secretion in the differentiated human B cell.

## Methods

### Reagents and cell culture

Recombinant human IL-6 was purchased from GenScript (Piscataway, NJ). Cannabinoid CB2 inverse agonist SR144528 and CB1 antagonist SR141716 were provided by NIH-NIDA-NDSP program. AM630 (CB2 inverse agonist) and HU308 (CB2 agonist) were purchased from Cayman Chemical (Ann Arbor, MI). Human IgM ELISA Kit (Immunology Consultants Laboratory, Inc., Portland, OR) was used according to manufacturer’s recommendations. ^3^H-Thymidine (46.5 Ci/mmol) was purchased from PerkinElmer (Boston, MA). TPA and LPS were from Sigma-Aldrich (Saint Louis, MO).

The human SKW 6.4 cell line was obtained from American Type Culture Collection. Cells were cultured in RPMI-1640 medium with 10% heat-inactivated fetal bovine serum (FBS), 2 mmol/L glutamine, and 100 U/mL penicillin–streptomycin (Sigma-Aldrich) at 37°C with 5% CO_2_. For the experiments, SKW 6.4 cells (5 × 10^4^/ml) were placed in 96-well flat-bottomed microtiter plates (200 μl/well) and were incubated with or without IL-6, SR144528 and the other agents for various times at 37°C. CB2 ligands were prepared in dimethyl sulfoxide (DMSO) stock solutions of 50 mM and diluted by medium before application. For all the cell cultures with CB2 ligands, the final concentrations of DMSO were always equal or less than 0.05%. Control cells were also treated with an equivalent amount of DMSO without the drugs.

### Determination of IgM secretion

Supernatants of SKW 6.4 cell cultures were harvested by centrifugation for the IgM enzyme-linked immunosorbent assay (ELISA). The IgM levels were assayed by sandwich ELISA following the kit manufacturer’s instruction. Briefly, each supernatant sample containing IgM was added into the well of the microtiter plates that have been coated with anti-human IgM antibodies. The plates were incubated at room temperature for 1 hr. After washing, anti-IgM antibody conjugated with horseradish peroxidase was added to the well and the plates were allowed to incubate for another 0.5 hr. After washing, citrate buffer (pH 3.3) containing the chromogen-substrate solution (3,3’5,5’-tetramethybenzidine and hydrogen peroxide) was added and incubated for 10 min in the dark. The optical densities at 450 nm were measured and the quantity of IgM in the test sample was interpolated from the standard curve constructed from the standards (human IgM calibrator) and corrected for sample dilution.

### Cell proliferation assay (^3^H-thymidine incorporation)

Growth of SKW 6.4 cells was measured by the ^3^H-thymidine incorporation method [23]. The cells were cultured in 96-well culture plates in RPMI-1640 medium containing 10% FBS with or without drugs for 72 hours at 37°C with 5% CO_2_. Cells were pulsed with 1 mCi/well ^3^H-thymidine during the last 8 hours of culture, harvested onto glass fiber filter mats (Wallac) with an automatic cell harvester. The radioactivity was determined by using a TopCount NXT scintillation counter (PerkinElmer).

### Real-time RT-PCR analysis

Total RNA was extracted with Trizol Reagent from Invitrogen (Invitrogen, Carlsbad, CA). The cDNA was synthesized from 1.0 μg of total RNA with the random hexamer primers and SuperScript RT III enzyme (Invitrogen), according to the manufacturer's protocol. SYBR Green-based real-time PCR was performed with the ABI 7300 Real-Time PCR System (Applied Biosystems, Foster City, CA). Data were normalized against the control of GAPDH signals.

The sequences for PCR primers were:

BCL6 forward 5’-GATGAGATTGCCCTGCATTT-3’ and reverse 5’-TTCTTCCAGTTGCAGGCTTT-3’.

PAX-5 forward 5’-GACGACATGAAGGCCAATCT-3’ and reverse 5’-TACTGAGGGTGGCTGTAGGG-3’.

GAPDH forward 5’-GAAGGTGAAGGTCGGAGT-3’ and reverse 5’-GAAGATGGTGATGGGATTTC-3’.

### SDS-PAGE and Western blotting

Western blotting was conducted as previously described [24]. Briefly, cells were harvested, lysed with radioimmunoprecipitation assay buffer (Pierce) containing phosphatase and protease inhibitors (Halt Protease Inhibitor Cocktail Kit; Pierce). Lysates were analyzed by SDS-PAGE and transferred to a polyvinylidene fluoride membrane. Following probing with specific primary antibodies plus horseradish peroxidase-conjugated secondary antibody, the protein bands were detected using SuperSignal West Pico Chemiluminescent Substrate (Pierce).

### Statistical analysis

Data values are expressed as mean ± S.D. Statistical differences were determined by Student *t* test. Results were considered significantly different for P < 0.05.

## Results and discussion

### IL-6 induces IgM secretion in a concentration-dependent manner

**Figure 1:**
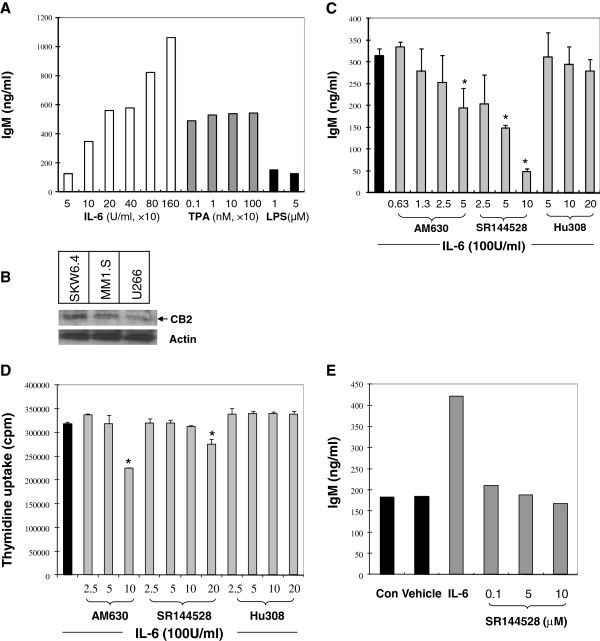
**Effects of CB2 ligands on IL-6-induced IgM production in SKW6.4 cells. (A)** IL-6, but not TPA or LPS, stimulated IgM secretion in a concentration dependent manner. SKW 6.4 cells were incubated for 4 days with the indicated concentrations of IL-6 (white bar), TPA (grey bar) or LPS (black bar). The secreted IgM was determined by ELISA. Background IgM secretion was 119 ng/ml and was subtracted from the IL-6-induced secreted IgM shown. **(B)** Expression of CB2 receptor in SKW 6.4 and human B malignancy myeloma cells MM1.S and U266. **(C)** Modulation of IL-6-induced IgM secretion by CB2 ligands. SKW 6.4 cells were incubated for 4 days with CB2 ligands (μM) plus IL-6 (100 U/ml in 0.04% DMSO). The secreted IgM was determined by ELISA. Background IgM secretion was 176 ng/ml and was subtracted from the IL-6-induced secreted IgM shown. Results were expressed as mean ± SD of three assays. **(D)** Effects of CB2 ligands on SKW6.4 cell proliferation. SKW 6.4 cells were incubated for 4 days with CB2 ligands plus IL-6 (100 U/ml in 0.04% DMSO). Cell DNA synthesis was determined with ^3^H-Thymidine uptake assay. **(E)** SKW6.4 cells were exposed to IL-6 (100 U/ml), SR144528 (0.1-10 μM) or vehicle control (0.05% DMSO, v/v) for 4 days. The untreated wells were used as control (Con). The secreted IgM was determined by ELISA. A representative experiment is shown. *P < 0.05 compared with IL-6 treatment alone.

Upon stimulation with IL-6, 12-*O*-tetradecanoylphorbol-13-acetate (TPA) or lipopolysaccharide (LPS), B cells are activated, rapidly proliferate and initiate differentiation processes leading to increased antibody secretion, e.g., immunoglobulin M (IgM) [19,21,22,25,26]. To determine which stimulator was most efficient for the present study, we treated SKW 6.4 cells with rhIL-6 (50-1600 U/ml), TPA (1-1000 nM) or LPS (1-5 μM) for 4 days. Among the three stimulators, only IL-6 raised the IgM secretion in a concentration-dependent manner, whereas TPA and LPS both could increase immunoglobulin production but without clear concentration responses over these ranges of concnetration (Figure 1A). We speculate that the different responses among the three stimulators may have resulted from their diverse downstream signal activation [19,21,27]. For the well-controlled study below, we chose to use IL-6 as the inducer of IgM secretion in SKW 6.4 cells.

### Inhibition of IL-6-triggered IgM production by CB2 inverse agonists

CB2 is primarily expressed in B plasma cells [3]. To determine CB2 gene expression in SKW 6.4 cells and assure the action of CB2 ligands, we first measured CB2 expression level by using immunoblot assay. As shown in Figure 1B, CB2 expression was detected in human SKW 6.4 cells and the positive controls, human malignancy plasma myeloma cells (MM1.S and U266).SKW 6.4 cells were next treated with three CB2 ligands (agonist HU308 and inverse agonists AM630, SR144528) in the presence or absence of IL-6 and IgM levels in the culture supernatant were measured. Strikingly, it was not the agonist, but the CB2 inverse agonists that reduced IL-6-induced IgM secretion in a concentration-dependent fashion (Figure 1C). The inhibitory effect of SR144528 was indicated by 100% and 160% decreases in IgM production at 5 μM and 10 μM, respectively, relative to cells treated with IL-6 alone. AM630 also showed inhibitory effects; whereas the agonist HU308 failed to inhibit IL-6-induced IgM production in the range of the tested concentrations (5-20 μM) (Figure 1C).

To rule out the possibility that the modulating effects of CB2 ligands on IgM production resulted from inhibition of cell proliferation, we treated the SKW 6.4 cells with various concentrations of the ligands for three days in the presence of IL-6. As shown in Figure 1D, treatment of SKW 6.4 cells with inverse agonists (AM630 below 5 μM, SR144528 below 10 μM) or agonist (HU308 below 20 μM) has no inhibitory effects on the growth of SKW 6.4 cells, as determined by the ^3^H-thymidine incorporation assay. These results indicate that the ligands may affect IL-6 signal transduction and IgM production independent of any effect on cell proliferation. In addition, SKW 6.4 cell culture without IL-6 also expressed a basal level of IgM. To see whether CB2 ligand SR144528 exhibits any modulation of the spontaneous IgM secretion, we treated SKW 6.4 cells with SR144528 only (0.1-10 μM) in the absence of IL-6 for 4 days. Data shown in Figure 1E revealed that exposure to SR144528 only slightly disturbed the IgM autocrine production relative to control vehicle. This suggests that SR144528 specifically influences IL-6-induced IgM production.

The differentiation of B cells into IgM-secreting plasma cells upon IL-6 stimulation is interrupted by the CB2 antagonist in SKW 6.4 cells. These effects are not derived from inhibition of cell growth. Since CB2 can positively regulate B cell immunity to respond to foreign antigens resulting in the early production of IgM, and the production of antigen-specific IgM is inhibited in CB2(-/-) mice [28], our data then suggest that CB2 antagonism may display effects on IgM production similar to that seen in a CB2 gene deletion.

### CB2 agonists reverse the inhibitory effect of SR144528 on IL-6-induced IgM secretion

**Figure 2:**
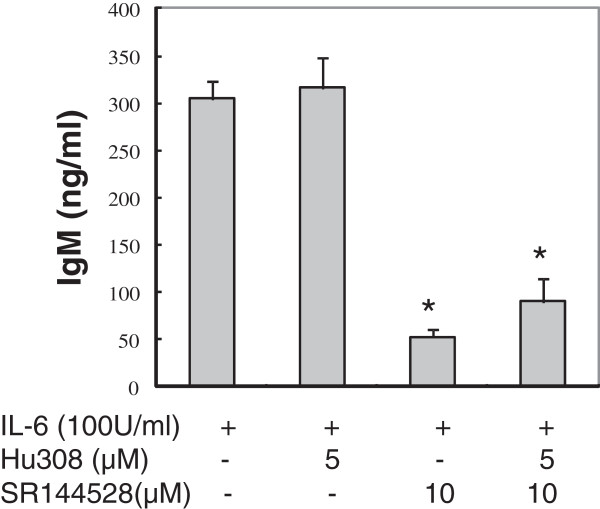
**CB2 agonist reverses the inhibitory effect of SR144528 on IL-6-induced IgM secretion.** SKW 6.4 cells were exposed to CB2 selective agonist HU308 (5 μM), SR144528 (10 μM) or their combination for 4 days in the presence of IL-6 (100 U/ml). IgM in the culture supernatants was measured by ELISA. Background IgM secretion was 126 ng/ml and was subtracted from the IL-6-induced secreted IgM shown. Results were expressed as mean ± SD of three assays. *P < 0.05 compared with the control (IL-6 treated alone).

To confirm the CB2 receptor-mediated inhibitory effects on IL-6-induced IgM production, we pretreated SKW 6.4 cells with CB2 specific agonist prior to exposure to SR144528. We observed that pretreatment with CB2 specific agonist HU308 (5 μM) prior to treatment with antagonist SR144528 could reverse the inhibitory effect of SR144528 on IL-6-stimulated IgM secretion (*P* = 0.06, Figure 2). Higher concentration of HU308 (10 μM) failed to further reverse the effects of SR144528 (data not shown). Treatment with HU308 without SR144528 at concentrations used in this study had no significant effects on IL-6-stimulated IgM secretion (Figure 2) and cell viability (Figure 1D). Hence this inhibitory effect on IgM production may be mediated, in part at least, by the peripheral CB_2_ receptor.

### Transcriptional regulation of IgM production

**Figure 3:**
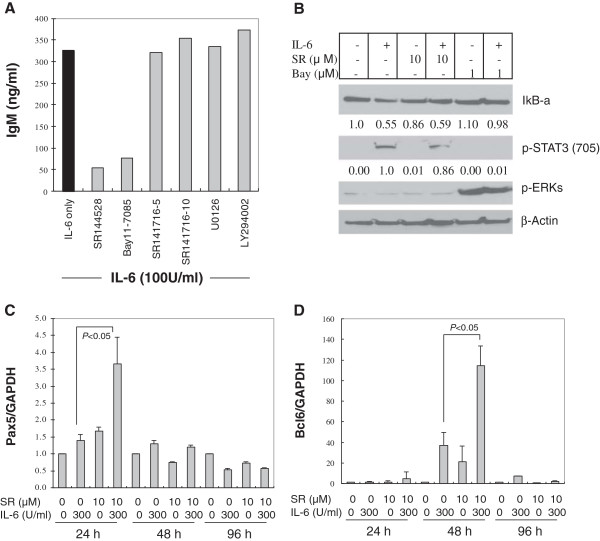
**Modulation of STAT3, but not NF-κB and PI-3 K and ERK/MEK, is involved in the inhibition of IL-6-induced IgM secretion by SR144528. (A)** SKW 6.4 cells were pretreated with CB2 antagonist SR144528 (10 μM) or CB1 antagonist SR141716 (5 or 10 μM) or the indicated inhibitors (Bay11-7085, 0.01 μM; U0126: 15 μM; LY294002, 15 μM) for 30 min, followed by IL-6 (100 U/ml) exposure for 4 days. IgM in the culture supernatants was measured by ELISA. Background IgM secretion was 99 ng/ml and was subtracted from the IL-6-induced secreted IgM shown. **(B)** SKW 6.4 cells were pretreated with SR144528 (SR) or Bay11-7085 (Bay) for 30 min, followed by IL-6 (300 U/ml) stimulation for another 45 min. After harvest, the whole-cell lysates were subjected to Western blot analysis and detected with indicated antibodies. β-Actin served as loading control. The numbers below the IκB-α and p-STAT3 (705) bands indicated the normalized ratios of the target band and their respective β-Actin. **(C-D)** SKW 6.4 cells were treated with IL-6 in the absence or presence of SR144528 (SR) for 24, 48 or 96 hrs, respectively. Total cellular RNA was extracted with Trizol Reagent and reverse transcribed into cDNA by using SuperScript RT III enzyme. SYBR Green-based real-time PCR for each gene was performed and the results were normalized against the control of GAPDH signals. Final concentrations of DMSO in the tested wells were equal or less than 0.05%.

NF-κB inhibition prevents Igκ gene expression and perturbs the assembly of IgM on cell surfaces, which marks the differentiation of immature B cells [29]. IL-6 may lead to NF-κB activation through its biological interplay of phosphorylated STAT3 signal crosstalk with NF-κB [30,31]. In this study, we found that a NF-κB specific inhibitor Bay11-7085 markedly inhibited the stimulatory effect of IL-6 on IgM secretion at the concentration of 0.01 μM (Figure 3A). Bay11-7085, at the concentration of 0.01 μM, had no inhibitory effect on the cell viability (data not shown). To study the effect of IL-6 on the NF-κB pathway, we used a higher concentration of IL-6 (300 U/ml) to treat the cells for a shorter time (45 min) and detected the IκBα (a cytoplasmic inhibitor protein of NF-κB) degradation. Although Bay11-7085 could inhibit IL-6-induced IκBα degradation, SR144528 pretreatment failed to significantly reverse the degradation (Figure 3B). This demonstrated that SR144528 and Bay11-7085 may not work in the same pathway on IL-6-induced IgM production in SKW 6.4 cells.

Interestingly, SR141716, a specific CB1 receptor antagonist, failed to show any inhibition of IL-6-induced IgM production in the same concentration range as CB2 antagonist SR144528 or AM630 (Figure 3A). This suggests that CB2, but not CB1 antagonism, may be primarily involved in the inhibition of IL-6-induced IgM secretion in the present cellular system. Together with the demonstration that human B cells display large amounts of CB2 receptor mRNA [3], the above results led us to assume that the inhibition activity observed on plasma cells could be mediated mainly through the CB2 receptor.

Since phosphatidylinositol 3-kinase (PI3K) and ERK/MAPK are all involved in IL-6 signaling and have been implicated as important mediators of B-cell activation and differentiation signals [31,32], we next asked whether they play roles in IL-6-induced IgM secretion in SKW 6.4 cells. We found that PI3K inhibitor LY294002 and MEK/ERK inhibitor U0126 failed to inhibit the IL-6 effect on IgM production (Figure 3A). In our cellular system, IL-6 had no significant activation effect on ERK kinase (Figure 3B). These data suggest that activation of PI3K and MEK/ERK pathways by IL-6 may not be associated with IgM secretion in SKW 6.4 cells. Our data are consistent with a previous report, in which IL-6 was shown to activate ERK, but the blockade of MEK/ERK had only a minimal effect on IL-6–induced IgM secretion in human BCWM.1 cells [33].

IL-6 is able to transduce intracellular signaling through a receptor complex called IL-6R but the downstream IL-6/PI3K and IL-6/ERK pathways have not yet been sufficiently addressed. Unlike TPA, IL-6 has been demonstrated as inactive to stimulate ERK-2 in SKW6.4 cells, but IL-6 can activate ERKs in another human B cell line, AF-10 [34]. We may therefore conclude from our data and literature reports that ERK status plays inessential role in IL-6-induced IgM secretion in SKW6.4 cells.

Phosphorylation of STAT3 is critical for B cell differentiation into immunoglobulin-secreting plasma cells [35,36]. Down-regulation of STAT3 can reduce IgM secretion [37]. As shown in Figure 3B, IL-6 strikingly induced STAT3 phosphorylation after 30 min exposure, whereas SR144528 pretreatment could moderately inhibit IL-6-raised STAT3 phosphorylation (Figure 3B). IκB kinase inhibitor Bay11-7085 could block IL-6-mediated STAT3 activation, supporting the existence of a link between NF-κB and STAT3 pathways in the context of IL-6 stimulation [30,31]. Pretreatment with SR144528, however, had no effect on IL-6-induced NF-κB activation, ruling out the possibility that SR144528 may inhibit IgM through the NF-κB pathway.

Besides the above kinases, several key regulatory proteins in the B-cell transcriptional network have been identified, with two coupled mutually repressive feedback loops among the three transcription factors: B-cell lymphoma 6 (Bcl-6), B lymphocyte-induced maturation protein 1(Blimp-1), and paired box 5 factor (Pax5) forming the core of the network [38,39]. Pax5 negatively regulates a set of genes associated with antibody secretion by plasma cells such as J chain, IgH and X-box binding protein 1 (XBP-1). Thus overexpression of Pax5 leads to increased cell proliferation and suppression of Ig synthesis [38]. By RT-qPCR assay, we found that Pax5 and Bcl-6 mRNA levels were dramatically increased by SR144528 treatment in IL-6-treated SKW 6.4 cells (Figure 3C-D). The time sequence showed that Pax5 was transactivated before Bcl-6, suggesting the early transient upregulation of Pax5 by SR144528 initiated the inhibition of IL-6 effect on IgM. Transactivation of Bcl-6 would be expected to continue to suppress plasma cell differentiation through negatively targeting the Blimp-1 gene, thus impeding differentiation to secretion. Other lines of evidence in the literature support our results. Bcl-6-/- mice have elevated antibody response and increased Ig-secreting cells [40]. In addition, decreased Blimp-1 transcription and upregulation of Pax5 inhibit B-cell differentiation into antibody-secreting plasma cells [38].

## Conclusion

In summary, we found that CB2 inverse agonists inhibited IL-6-induced immunoglobulin in human SKW 6.4 cells through regulation of STAT3 pathway and relevant transcriptional factors including Bcl-6 and Pax5. Our study corroborates the finding that cannabinoids exhibit important regulation in the early humoral immune response through CB2 specific expression in B cells. Interestingly, SR144528 had no effect on the constitutive secretion of IgM but effectively diminished IL-6-induced immunoglobulin production. By contrast, the CB_2_ receptor agonist HU308 (5-20 μM) did not itself induce an inhibitory effect; however, it reduced the inhibitory effect of SR144528 on IL-6-induced IgM. The findings on the regulatory function of the CB2 ligands in this study differs from the one described by Eisenstein et al. [7] because our use of human cells requires IL-6 stimulation. Whether CB2 inverse agonists negatively regulate pathologic globulin production (such as autoimmune Ab or paraprotein in myeloma) warrants further study.

## Competing interests

The authors declare that they have no competing interests.

## Authors’ contributions

RF designed and performed research, analyzed data, wrote and revised the paper; CAM participated in the conception design and coordination and helped to draft the manuscript; XQX designed the research, analyzed data, wrote and revised the paper. All authors read and approved the final manuscript.

## Pre-publication history

The pre-publication history for this paper can be accessed here:

http://www.biomedcentral.com/2050-6511/15/30/prepub
